# Prevalence of hepatopancreatic injury and clinical outcomes in patients with COVID-19 in USA

**DOI:** 10.1186/s12245-021-00393-2

**Published:** 2021-11-06

**Authors:** Vaibhav Rastogi, Ranjit Banwait, Devina Singh, Hale Toklu, Lexie Finer, Dipendra Parajuli, Latha Ganti

**Affiliations:** 1 HCA Healthcare Graduate Medical Education, Nashville, Tennessee USA; 2 Envision Physician Services, Plantation, Florida USA; 3 Department of Clinical Sciences, University of Central Florida College of Medicine, Florida Orlando, USA; 4 Department of Gastroenterology, University of Louisville, Louisville, Kentucky USA

**Keywords:** Liver, Pancreas, Mortality, SARS-CoV2

## Abstract

**Background:**

(1) To determine the prevalence of hepatopancreatic injury in coronavirus disease 2019 (COVID-19) patients. (2) To correlate hepatopancreatic injury in COVID-19 with mortality, disease severity, and length of stay in this cohort.

**Results:**

Forty-five thousand three hundred sixty patients were included in the analysis, 62.82% of which had either hepatic or pancreatic injury. There was a significant upward trend in transaminases, alkaline phosphatase, prothrombin time, bilirubin, lactate dehydrogenase, and lipase and a downward trend in albumin with an increase in disease severity. COVID-19-positive patients with hepato-pancreatic injury have a significantly higher mortality (OR 3.39, 95%CI 3.15–3.65) after controlling for the differences in age, sex, race/ethnicity, liver cirrhosis, and medication exposures. They also have increased disease severity (OR 2.7, 95%CI 2.5–2.9 critical vs mild/moderate; OR 1.4, 95% CI 1.3–1.5 severe vs mild/moderate) and longer hospital length of stay (2 days).

**Conclusion:**

COVID-19 can cause liver injury. Mortality, disease severity, and hospital length of stay are increased in COVID-19 patients with hepatopancreatic injury.

**Graphical Abstract:**

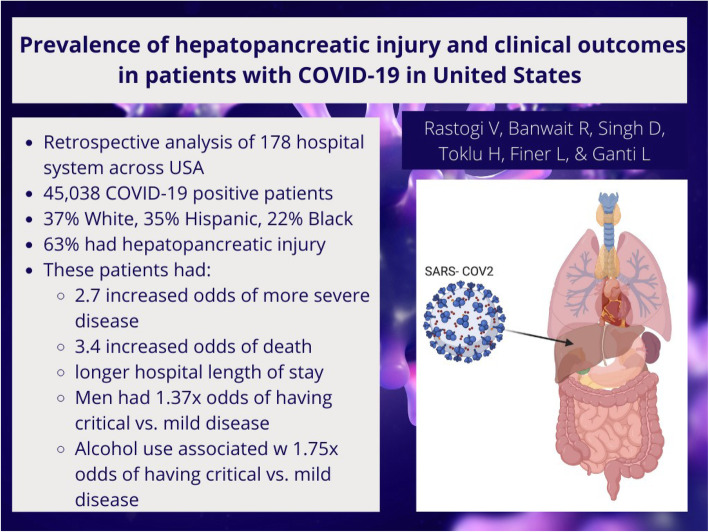

## Background

The first case of coronavirus disease 2019 (COVID-19) in the USA was noted on January 20, 2020, in Washington state. Since then, it has spread exponentially, resulting in more than 34 million cases and 613,089 deaths as of July 18, 2021. COVID-19 affects multiple organ systems, including the gastrointestinal system.

Liver injury has been associated with major pathogenic coronavirus including severe acute respiratory syndrome coronavirus (SARS-CoV), Middle East respiratory syndrome coronavirus (MERS-CoV), and the newly emergent SARS-CoV-2, which causes COVID-19 [1]. Elevation in the levels of markers of liver injury including alanine aminotransferase (ALT) and aspartate aminotransferase (AST) was noted in 4–39% and 4–58% of the COVID-19-positive patient populations, respectively. Alkaline phosphatase (AlkP) elevations were observed in 2–5% of the cohorts [2]. Zhang et al. reported hypoalbuminemia in 55% of 115 COVID-19-positive patients [3]. Hyperbilirubinemia was observed in 1–18% of the patient pool [2]. Liu et al. reported the incidence of pancreatic injury in 1–2% of mild and 17% of severe cases [4]. Hepatopancreatic injury appears to be significantly more common among those with severe infection [2, 4]. Here, in our retrospective cohort study, we attempt to assess the association of hepato-pancreatic derangement with COVID-19 infection as well as its impact on patient prognosis.

## Methods

### Study design

This is a retrospective observational cohort study from HCA Healthcare data. HCA Healthcare is a large heath care system that includes 178 hospitals across the USA.

### Data collection and review

All patients who presented with COVID-19 (ICD10 U07.1) at one of the HCA hospitals nationwide between January 1, 2020, and September 1, 2020, were included in this study. SARS-CoV-2 was confirmed with polymerase chain reaction (PCR) testing of a nasopharyngeal or oropharyngeal swab. Data was extracted from the enterprise electronic medical records by a research analyst who created a de-identified data set. All study records were kept in a password-protected study folder on a closed, enterprise-owned network.

### Data elements and outcomes

Data elements included patient demographics, comorbidities, home medication, vitals and laboratory tests conducted during hospitalization, inpatient diagnoses, inpatient medications, treatments, procedures including invasive mechanical ventilation, length of hospital stay, and mortality.

The primary outcome was mortality, which included all-cause death or hospice discharge. Secondary outcomes included (1) severity of COVID-19, with mild/moderate disease defined as the highest level of care being medical floor, critical disease defined as the highest level of care being intensive care unit (ICU) and requiring mechanical ventilation and/or vasopressor support, and severe disease defined as the highest level of care being ICU but not meeting criteria for critical disease; and (2) length of hospital stay. These outcomes were recorded for patients who completed their hospital course at the end of the study period (September 1, 2020).

Pancreatic injury is defined as a lipase value of above 400 U/L. Hepatic injury is defined as either having a AST value above 37 U/L or a ALT value above 61 U/L. We also evaluated AlkP, prothrombin time (PT), lactate dehydrogenase (LDH), bilirubin, and albumin, but these were not used to define hepato-pancreatic injury. All the labs have been restricted to cut off the top 1% of values to reduce outliers, and the maximum values for all labs except albumin were taken respectively within the first 10 days of a patient’s admission. Minimum values were taken for albumin as liver injury results in a decrease in albumin. We used 10 days as a cutoff because at day 10 cytokines in moderate disease start declining whereas in severe disease, they remain elevated [5].

### Statistical analysis

All analysis in this study was completed using SAS (Version 9.4). *P* values were assessed at the 95% confidence level (*α*=0.05).

Demographic and laboratory tests on the first day of admission were summarized using percentage for categorical variables and mean (standard deviation) for continuous variables. The chi square test was used to assess e rate of positive COVID-19 screenings and inpatient mortality. The length of stay was assessed with a two-sample Wilcoxon rank sum. The lab values among groups in regard to the disease severity were compared by ANOVA.

## Results

The study population consisted of 45,360 COVID-19-positive patients, of which 52% were male. Sixty-three percent had either hepatic or pancreatic injury. The ethnoracial composition of the cohort was 37% White, 35% Hispanic, 22% African American, and 6% other. The mean age was 61.5 years. Majority (57%) of patients were admitted to the medical floor, along with 29% that were admitted to the ICU. At the time of initial presentation, 756 (1.7%) and 1355 (3%) patients had nausea/vomiting and diarrhea respectively. Hepatic injury was seen in 28,310 (62.4%) patients, whereas pancreatic injury was seen in 825 (1.8%) patients. Pancreatitis was diagnosed in 366 (0.8%) patients, and cholelithiasis/cholecystitis was diagnosed in 943 (2%). Liver cirrhosis was present in 2171 patients (4.8%) and only 3% of the study population were alcoholics (Table 1).

**Table 1:** Study population demographic data and characteristics

	*N*	%
Patient population	45,360	100
Gender		
Female	21,826	48.12
Male	23,534	51.88
Race/ethnicity		
Black	9988	22.02
Hispanic	15,968	35.2
White	16,860	37.17
Other	2544	5.61
Hepatic injury	28,310	62.41
Pancreatic injury	825	1.82
Hepatic and/or pancreatic injury	28,495	62.82
Chronic liver disease (liver cirrhosis)	2171	4.79
Alcohol use	1386	3.06
Pancreatitis	366	0.81
Cholelithiasis/cholecystitis	943	2.08
Nausea and vomiting	756	1.67
Diarrhea	1355	2.99
Antiviral use	2800	6.17
Antibiotic use	19,109	42.13
Corticosteroids use	5078	11.19
Mortality	6604	14.56
Disease severity		
Mild/moderate	6484	14.29
Severe	25,849	56.99
Critical	13,027	28.72

The mean values for AST, ALT, AlkP, and lipase in the hepato-pancreatic injury group were 85.4 U/L, 94.5 U/L, 109.5 U/L, and 226 U/L respectively. The means of total bilirubin, albumin, PT, and LDH in the same group were 0.8 mg/dL, 2.7 g/dL, 14, and 474 U/L, respectively. These values were significantly (*p*< 0.0001) higher from the means in the group that did not have hepato-pancreatic injury except for albumin that was significantly lower (Table 2).

**Table 2:** Difference in lab values between the study groups based on presence of hepatopancreatic injury (*t* test; *p*< 0.0001 for all labs)

Variable (mean, standard deviation)	Hepatopancreatic injury not present (*N* = 16,865)	Hepatopancreatic injury present (*N* = 28,495)
Alanine transaminase (ALT)	25.28, 10.89	85.38, 89.76
Aspartate transaminase (AST)	25.38, 7.23	94.53, 89.76
Alkaline phosphatase (AlkP)	89.96, 38.36	109.5, 61.38
Total Bilirubin	0.5875, 0.3565	0.8512, 0.6009
Albumin	2.98, 0.69	2.71, 0.72
Prothrombin time (PT)	13.35, 5.05	13.99, 5.30
Lipase	116.5, 79.04	226, 321.4
Lactate dehydrogenase (LDH)	283.3, 131.9	474.0, 257.2

Approximately 57% of COVID-19-positive patients were in the severe severity group (*n*=25,849), and 13,027 patients (29%) were critically ill. The severity of the disease significantly (*p*< 0.0001) increased with the age as expected; mean age was 63.5 for critically ill patients, whereas it was 53.5 for mild/moderate severity. Male gender was also significantly associated (*p*< 0.001) with increased disease severity (57.3% vs 45.6%, critical vs mild/moderate). Alcohol use was more commonly noted (*p*< 0.0001) in critically sick patients. Nausea/vomiting was more prevalent (*p*< 0.0001) in mild/moderate disease severity, whereas diarrhea was more common in critical patients (*p*=0.8). Although only 366 patients were diagnosed with pancreatitis, it was significantly (*p*< 0.0001) more common in critical patients (*n*=126). Similarly, cholelithiasis/cholecystitis was diagnosed more frequently in critical patients (*p*< 0.0001) (Table 3).

**Table 3:** Study population characteristics depending upon disease severity

Variable	Mild/moderate patients (*N* = 6484)	Severe patients (*N* = 25,849)	Critical patients (*N* = 13,027)
Age (mean, standard deviation)	53.50, 18.78	62.51, 17.45	63.51, 16.18
Sex (male)	2955 (45.57%)	13,107 (50.71%)	7472 (57.36%)
African American	1562 (24.09%)	5768 (22.31%)	2658 (20.40%)
Caucasian	1897 (29.26%)	9956 (38.52%)	5007 (38.44%)
Hispanic	2709 (41.78%)	8780 (33.97%)	4479 (34.38%)
Other race	316 (4.87%)	1345 (5.20%)	883 (6.78%)
Alcohol use	127 (1.96%)	790 (3.06%)	469 (3.60%)
Nausea/vomiting	179 (2.76%)	445 (1.72%)	132 (1.01%)
Diarrhea	187 (2.88%)	770 (2.98%)	398 (3.06%)
Pancreatitis diagnosis	21 (0.32%)	219 (0.85%)	126 (0.97%)
Cholelithiasis/cholecystitis diagnosis	66 (1.02%)	566 (2.19%)	311 (2.39%)

In the COVID-19-positive patients mean ALT (81.7 U/L) was significantly (*p*< 0.0001) higher in the critically sick patients in comparison to patients in the mild/moderate group (50 U/L) and severe group (57.1 U/L). Mean AST was also significantly (*p*< 0.0001) higher in the critical patients (96.6 vs 59.7 vs 49.8 U/L; critical vs severe vs mild/moderate). Similarly, mean values of other liver injury markers including AlkP, PT, LDH, and total bilirubin were significantly (*p*< 0.0001) increased in the critical severity group. Albumin levels were significantly lower (*p*< 0.0001) in the critical patients (2.41 vs 3.15 g/dL; critical vs mild/moderate). The levels of lipase were also significantly elevated (*p*< 0.0001) in the critical group vs mild/moderate group (mean 217 vs 145). Overall, the mortality was substantially higher (*p*< 0.0001) in the patients who were critically sick (35.1% vs 6.6% vs 4.8%; critical vs severe vs mild/moderate). Critical patients also had significantly longer (*p*< 0.0001) length of stay in comparison to severe and mild/moderate severity groups (13.7 vs 6.8 vs 2.8 days). These data are summarized in Table 4.

**Table 4:** Comparative analysis of the study population based on severity (ANOVA, *p*< 0.0001 for all variables)

Variable	Mild/moderate patients (*N* = 6484)	Severe patients (*N* = 25,849)	Critical patients (*N* = 13,027)
ALT (mean, standard deviation)	49.98, 53.77	57.12, 67.01	81.67, 99.55
AST (mean, standard deviation)	49.76, 51.32	59.69, 62.58	96.65, 106.8
AlkP (mean, standard deviation)	92.9, 45.88	97.45, 50.19	116.3, 64.19
Total bilirubin (mean, standard deviation)	0.6235, 0.414	0.7002, 0.4667	0.9219, 0.6702
Albumin (mean, standard deviation)	3.15, 0.69	2.94, 0.66	2.41, 0.66
PT (mean, standard deviation)	12.94, 3.99	13.45, 4.78	14.54, 6.06
Lipase (mean, standard deviation)	145.07, 165.63	183.45, 254.55	217.47, 329.47
LDH (mean, standard deviation)	353.57, 201.65	365.77, 196.79	529.81, 286.05
Mortality (*N*)	309 (4.77%)	1720 (6.65%)	4575 (35.12%)
Length of stay (days)	2.84, 4.45	6.76, 6.64	13.68, 11.71

*ALT* alanine transaminase, *AST* aspartate transaminase, *AlkP* alkaline phosphatase, *PT* prothrombin time, *LDH* lactate dehydrogenase

COVID-19-positive patients with hepato-pancreatic injury are 3.4 times more likely to die as compared to COVID-19-positive patients without hepato-pancreatic injury (OR 3.39, 95%CI 3.15–3.65) after controlling for the differences in age, sex, race/ethnicity, liver cirrhosis, and medication exposures (antivirals, antibiotics, and steroids). COVID-19-positive patients with hepato-pancreatic injury on an average have a length of stay two days longer than those without COVID-19-positive patients without hepato-pancreatic injury (*p*< 0.0001), maintaining all other predictors remain constant [age, sex, race/ethnicity, liver cirrhosis, and medication exposures (antivirals, antibiotics, and steroids)]. Hepato-pancreatic injury in COVID-19 patients can make them sicker. The odds of experiencing critical disease compared to mild/moderate disease is 2.7 times as likely for patients with hepato-pancreatic injury compared to those without hepato-pancreatic injury, assuming all the other variables are held constant (OR 2.7, 95%CI 2.5–2.9). The odds of experiencing severe disease compared to mild/moderate disease is 1.4 times as likely for patients with hepato-pancreatic injury compared to those without hepato-pancreatic injury, assuming all the other variables are held constant (OR 1.4, 95% CI 1.3–1.5). Male sex is associated with a significant increase in mortality, length of stay, and disease severity in COVID-19 patients (*p*< 0.0001), maintaining all other predictors remain constant. Similarly, patients with liver cirrhosis are also associated with significantly higher mortality, length of stay, and disease severity (*p*< 0.0001) (Table 5).

**Table 5:** Multivariate analyses of factors associated with disease severity, hospital length of stay, and mortality in study population (*N*=45,360)

Variables	Disease severity				Mortality		Length of stay	
	Critical vs mild/moderate		Severe vs mild/moderate					
	OR	95%CI	OR	95%CI	OR	95%CI	Coefficient	***P*** value
Hepato-pancreatic injury	2.710	2.532–2.901	1.398	1.320–1.481	3.389	3.150–3.646	2.004	<0.0001
Age	1.029	1.027–1.031	1.026	1.024–1.028	1.065	1.063–1.068	− 0.068	<0.0001
Sex (male vs female)	1.370	1.284–1.461	1.156	1.092–1.224	1.177	1.110–1.248	0.494	<0.0001
Alcohol use	1.750	1.418–2.160	1.540	1.267–1.873	0.982	0.817–1.181	1.787	<0.0001
Liver cirrhosis	1.941	1.620–2.327	1.798	1.516–2.133	1.321	1.163–1.500	0.889	<0.0001

## Discussion

In our study of 45,360 COVID-19-positive patients, hepatopancreatic derangement was observed in 28,495 patients. To our knowledge till date, this is the largest retrospective cohort study evaluating the hepatopancreatic injury in COVID-19 patients. Several studies have focused on the role of COVID-19 in hepatic injury [3, 6, 7]. Liver injury was seen in 62.4% of our patient population. This is less than the 78% incidence reported by Zhang et al. [6]. Their sample size was significantly smaller (0.2%, *n*=82) than ours. Huang et al. observed hepatic abnormalities in 31% of 41 patients admitted for COVID-19 infection with higher incidence in severe patients [7]. In contrast to hepatic injury, pancreatic injury was observed in only a small subset of patients (1.8%). Multiple studies have reported a mild increase in lipase (< 180 U/L) in a small percentage (12–17%) of COVID-19 patients [8, 9]. The smaller percentage in our study could be due to the higher cutoff value for lipase (400 U/L). Liu et al. reported 7.5% of severe patients who died had pancreatic injury. They also noted focal enlargement or dilation of pancreatic duct in these patients [4]. Wang et al. observed hepatic injury and pancreatic injury in 29% and 17% of patients respectively [10].

The magnitude of hepatopancreatic injury increased with an increase in disease severity. Critically sick patients have a significant elevation of transaminases, AlkP, PT, bilirubin, LDH, and lipase in comparison to patients with mild/moderate disease. This difference was also significant between ICU patients who required mechanical ventilation and/or vasopressor use and those who did not. Hypoalbuminemia was significantly worse in the critically sick population as compared to the other two groups. Guan et al. reviewed the clinical characteristics of 1099 COVID-19 patients in China and noted that increased levels of AST was found in 18% and 39.4% patients with non-severe disease and severe disease respectively. They also found that levels of ALT were elevated in 19.8% of non-severe diseases and 28.1% of severe disease patients [11]. Several meta-analyses also concluded that there is a higher incidence of liver injury in COVID-19 patients with severe disease as compared to non-severe disease [12, 13]. Barlass et al. pointed out that patients who require ICU admission also have a higher level of lipase [14].

We observed that COVID-19-positive patients who had hepato-pancreatic injury have higher mortality and severity of the disease. Lei et al. observed that an increase in ALT, AST, AlkP, and total bilirubin was associated with increased mortality in a retrospective cohort of 5771 COVID-19-positive individuals. Increase in AST was much more commonly associated with higher disease severity as well as confers the highest risk for death amongst all markers [15].

Hepatic injury in COVID-19 can be caused by multiple pathophysiologic mechanisms including direct virus-induced effects, immune system-mediated damage due to excessive inflammatory responses, and drug-induced injury. SARS-CoV2 can exert a direct cytopathic effect on liver resulting in hepatic injury. Postmortem biopsy of COVID-19 patient showed microvesicular steatosis, necrosis, and cellular infiltration in liver tissue. SARS-CoV2 binds to membrane-bound angiotensin-converting enzyme 2 (ACE2) receptor to enter the cells [16]. ACE2 receptors have a considerably higher expression on cholangiocytes (59.7%) as compared to hepatocytes (2.6%). Expression of ACE2 receptors on cholangiocytes is similar to that on type 2 alveolar cells [17]. Thus, COVID-19 can potentially cause liver injury of the same degree that is seen in the lungs. ACE2 receptors are also expressed on pancreatic cells (exocrine glands and islets) and this expression is mildly increased in contrast to the lungs [4].

Cytokine storm syndrome induced by COVID-19 is also likely to be blamed for hepatopancreatic injury. Several studies have reported elevated levels of hepatic enzymes and pro-inflammatory markers in association with severe cases of COVID-19 [2]. Multiple antiviral medications including Remdesivir, lopinavir, ritonavir, and corticosteroids can cause drug-induced hepatic injury. Other medications that can also cause hepatotoxicity include, hydroxychloroquine, acetaminophen, tocilizumab, and multiple antibiotics [16]. Corticosteroids and non-steroidal anti-inflammatory drugs can also cause drug-induced pancreatitis [18]. Liu et al. pointed that the abnormalities in the laboratory markers are likely due to the medical treatments rather than COVID-19 itself [19].

Chronic liver disease mainly liver cirrhosis, was present in 4.8% of our patient subset. We observed a significant increase in mortality, length of stay, and disease severity in patients who had underlying liver cirrhosis. Several studies have reported chronic liver conditions in 1–11% of COVID-19 patients with hepatic injury [2]. Kovalic et al. noticed a low (3%) prevalence of chronic liver disease in a metanalysis of 24,299 COVID-19 patients. They also outlined a noteworthy association of chronic liver disease with increased severity and mortality in COVID-19 patients [20]. SECURE (surveillance epidemiology of coronavirus under research exclusion) Cirrhosis Registry and European association for the study of the liver COVID-hep registry have been created to assess the effects of COVID-19 on patients with chronic liver disease and those with post-liver transplantations.

### Limitations

Study limitations include first, this is an observational retrospective cohort study, thus any associations found cannot be taken as a causal relationship between COVID-19 and hepatopancreatic injury. Second, oropharyngeal and nasopharyngeal swabs were used in COVID-19 detection and both tests have different sensitivity and specificity which might result in variation in a number of false positives or negatives. Third, the cutoffs for AST, ALT, and Lipase might have influenced the results.

## Conclusion

In summary, COVID-19 is associated with hepatic injury. It has a weak association with pancreatic injury. Hepatopancreatic injury is associated with higher mortality, disease severity, and length of stay in COVID-19 patients. It is essential for the clinicians to follow the liver function panel in the COVID-19 patients that are hospitalized as it helps in ascertaining the prognosis of this patient population. Emergency room physicians can take liver function panel into account to determine the severity of COVID-19 infection and whether the COVID-19 patient needs to be admitted into the hospital for further management.

## Data Availability

The data that support the findings of this study are available from HCA Healthcare but restrictions apply to the availability of these data, which were used under license for the current study, and so are not publicly available. Data are however available from the authors upon reasonable request and with permission of HCA healthcare.

## Abbreviations

COVID-19: Coronavirus disease 2019
SARS-CoV: Severe acute respiratory syndrome coronavirus
MERS-CoV: Middle East respiratory syndrome coronavirus
ALT: Alanine aminotransferase
AST: Aspartate aminotransferase
AlkP: Alkaline phosphatase
HCA: Hospital Corporation of America
IRB: Institutional Review Board
ICU: Intensive care unit
PT: Prothrombin time
LDH: Lactate dehydrogenase
ACE2: Angiotensin-converting enzyme 2
